# Hypoxia impairs adaptation of skeletal muscle protein turnover- and AMPK signaling during fasting-induced muscle atrophy

**DOI:** 10.1371/journal.pone.0203630

**Published:** 2018-09-13

**Authors:** C. C. de Theije, A. M. W. J. Schols, W. H. Lamers, D. Neumann, S. E. Köhler, R. C. J. Langen

**Affiliations:** 1 Department of Respiratory Medicine, NUTRIM School of Nutrition and Translational Research in Metabolism, Maastricht University Medical Center+, Maastricht, the Netherlands; 2 Department of Anatomy & Embryology, NUTRIM School of Nutrition and Translational Research in Metabolism, Maastricht University Medical Center+, Maastricht, the Netherlands; 3 Department of Pathology, CARIM School for Cardiovascular Diseases, Maastricht University Medical Center+, Maastricht, the Netherlands; University of Louisville School of Medicine, UNITED STATES

## Abstract

**Background:**

Hypoxemia in humans may occur during high altitude mountaineering and in patients suffering from ventilatory insufficiencies such as cardiovascular- or respiratory disease including Chronic Obstructive Pulmonary Disease (COPD). In these conditions, hypoxemia has been correlated to reduced appetite and decreased food intake. Since hypoxemia and reduced food intake intersect in various physiological and pathological conditions and both induce loss of muscle mass, we investigated whether hypoxia aggravates fasting-induced skeletal muscle atrophy and evaluated underlying protein turnover signaling.

**Methods:**

Mice were kept under hypoxic (8% oxygen) or normoxic conditions (21% oxygen), or were pair-fed to the hypoxia group for 12 days. Following an additional 24 hours of fasting, muscle weight and protein turnover signaling were assessed in the gastrocnemius muscle by RT-qPCR and Western blotting.

**Results:**

Loss of gastrocnemius muscle mass in response to fasting in the hypoxic group was increased compared to the normoxic group, but not to the pair-fed normoxic control group. Conversely, the fasting-induced increase in poly-ubiquitin conjugation, and expression of the ubiquitin 26S-proteasome E3 ligases, autophagy-lysosomal degradation-related mRNA transcripts and proteins, and markers of the integrated stress response (ISR), were attenuated in the hypoxia group compared to the pair-fed group. Mammalian target of rapamycin complex 1 (mTORC1) downstream signaling was reduced by fasting under normoxic conditions, but sustained under hypoxic conditions. Activation of AMP-activated protein kinase (AMPK) / tuberous sclerosis complex 2 (TSC2) signaling by fasting was absent, in line with retained mTORC1 activity under hypoxic conditions. Similarly, hypoxia suppressed AMPK-mediated glucocorticoid receptor (GR) signaling following fasting, which corresponded with blunted proteolytic signaling responses.

**Conclusions:**

Hypoxia aggravates fasting-induced muscle wasting, and suppresses AMPK and ISR activation. Altered AMPK-mediated regulation of mTORC1 and GR may underlie aberrant protein turnover signaling and affect muscle atrophy responses in hypoxic skeletal muscle.

## Introduction

A decrease in blood oxygen tension and saturation (hypoxemia) occurs in response to hypobaric hypoxia during high altitude mountaineering [1] and in patients suffering from ventilatory insufficiencies as may occur in cardiovascular [2] or respiratory disease including Chronic Obstructive Pulmonary Disease (COPD) [3–5]. In these conditions, hypoxemia has been correlated to decreased food intake [6–8] and both induce loss of skeletal muscle mass. Skeletal muscle wasting in patients with COPD is a predictor of mortality and significantly increases disease burden [9, 10]. Skeletal muscle atrophy in response to fasting has been documented in humans [11] as well as experimental animal models [12]. Weight loss and muscle wasting are prominent features in COPD patients suffering from chronic hypoxemia [5], and rats [13, 14] and mice [15] with hypoxemia induced by ambient hypoxia. Weight loss and muscle wasting have also been associated with short-term hypoxemic episodes during acute disease exacerbations in COPD [16–18]. An inverse correlation between the degree of weight loss prior to admission and resting arterial oxygen tension at hospitalization for acute exacerbations was reported by Vermeeren et al [19]. Moreover, upon admission mean dietary intake was decreased by 40%, and only normalized after 4 days hospitalization [7].

Muscle mass maintenance requires a balance between protein synthesis and protein degradation. Increased protein degradation and reduced synthesis results in an imbalanced muscle protein turnover signaling and subsequent muscle wasting. The ubiquitin 26S-proteasome system (UPS) and autophagy lysosomal pathway (ALP) play a major role in the degradation of muscle protein. Rate-determining enzymes of proteasomal protein degradation are E3 ubiquitin ligase tripartite motif containing 63 (TRIM63, MURF1) and F-box protein 32 (FBXO32, ATROGIN-1). Increased expression of these effectors of the UPS [20] has been associated with skeletal muscle loss induced by fasting [21–25] and hypoxia [15, 26]. The expression of the ALP markers BCL2/adenovirus E1B 19kDa protein interacting protein 3 (BNIP3) and microtubule-associated protein 1 light chain-3B (MAP1LC3B) are also increased by fasting and hypoxia [27–29]. BNIP3 can be regulated by glucocorticoid receptor (GR)-dependent transcriptional regulators, such as Krüppel-like factor 15 (KLF15) [30] and Forkhead box O1 (FOXO1), which are involved in proteasomal and autophagy lysosomal protein degradation [28, 31, 32]. GR also regulates protein synthesis through KLF15 [30] and DNA-damage-inducible transcript 4 protein (DDIT4, a.k.a. REDD1) expression [33]. REDD1 controls protein synthesis through inhibition of mammalian target of rapamycin complex 1 (mTORC1) by activation of tuberous sclerosis complex 2 (TSC2) [34]. TSC2 activity and mTORC1 activity can further be controlled by protein kinase B (PKB, a.k.a. AKT1) and AMP-activated protein kinase (AMPK) [35]. AMPK not only regulates mTORC1 activity but is also involved in REDD1 expression [36], regulation of autophagy by UNC51-like kinase 1 (ULK1) [37] and GR mediated protein degradation signaling [38]. In addition to AMPK, hypoxia and starvation also activate the Integrated Stress Response (IRS), resulting in inhibition of protein synthesis and increasing autophagic flux [39].

Since hypoxemia and reduced food intake intersect in various physiological and pathological conditions and both induce loss of muscle mass, we hypothesized that hypoxia aggravates fasting-induced skeletal muscle atrophy, characterized by an additive shift of protein turnover towards proteolysis signaling. To test this hypothesis, mice were exposed to hypoxia for 12 days, and subsequently subjected to 24 hrs of fasting. A normoxic group that was pair-fed to the hypoxic animals was included to discern effects of hypoxemia from hypoxia-induced reductions in food intake prior to the fasting period. Protein turnover signaling pathways were assessed in the gastrocnemius muscle homogenates by RT-qPCR and Western blot analysis.

## Methods

### Animals and tissue collection

The mouse studies were carried out according to a protocol approved by the Institutional Animal Care Committee of Maastricht University. 12 week old male C57BL6/J mice were randomly divided into 3 groups: normoxia (N), normobaric hypoxia (H) and normoxia with pair-feeding to the hypoxia group (PN). All mice were housed in experimental chambers at 21°C with a 12-hour dark/light cycle. Mice received standard chow (V1534-000 Ssniff R/M-H, Ssniff Spezialdiäten) and water *ad libitum*. After 5 days of acclimatization, normoxic and pair-fed mice were exposed to 21% oxygen and hypoxic groups to normobaric hypoxia. Using the proOX system P110 (BioSpherix), ambient oxygen was reduced in a stepwise manner to 12% (day 1), 10% (day 2), and finally 8% (61 mmHg) on day 3, which was then maintained for the remainder of the experiment. To assess the effects of reduced food intake during hypoxia, PN animals received daily the amount of food consumed by the hypoxic mice at start of their active period (15:00). After 12 days, body weights and food-intake had been stable for at least 5 days. For the 24-hour-fasting protocol, mice were placed in clean cages without food but water *ad libitum* and each group consisted of 8 mice. Directly after 24 hrs fasting mice were killed. Gastrocnemius muscle was collected and frozen immediately in liquid nitrogen for further analysis. Muscle weights were corrected for tibia length.

### RT-qPCR

Total RNA was isolated using TRI REAGENT^™^ (Sigma-Aldrich) and further purified by precipitation with 2 M LiCl. cDNA synthesis was performed with random hexamer primers on denatured RNA using the Transcriptor First Strand cDNA Synthesis Kit (Roche). Real-time quantitative PCR (RT-qPCR) was performed in the iQ5 thermal cycler (Biorad) using the qPCR SyBr Green Fluorescein Mix (Abgene) and LinRegPCR software was used to analyze the data. qPCR primers were designed using Primer Express 2.0 software (Applied Biosystems) and ordered from Sigma Genosys (Table 1). All primers were intron-spanning to avoid contamination of the amplification products with genomic DNA. Expression of genes was normalized to *18S* ribosomal RNA. RT-qPCR data are normalized to basal mRNA concentrations under normoxic conditions without fasting (day 0).

**Table 1:** Sequences of primers used for RT-qPCR to assess the expression of the indicated genes.

Gene	NCBI nr.	Forward primer (5’ to 3’)	Reverse primer (5’to 3’)
18S	NR_003278.1	AGTTAGCATGCCAGAGTCTCG	TGCATGGCCGTTCTTAGTTG
Gr	NM_008173	CGCCAAGTGATTGCCGC	TGTAGAAGGGTCATTTGGTCATCCA
Glul	NM_008131.3	GGCCATGCGGGAGGAGA	GGTGCCTCTTGCTCAGTTTGTCA
Foxo1	NM_019739.3	AAGAGCGTGCCCTACTTCAAG	CCATGGACGCAGCTCTTCTC
Klf15	NM_023184.3	TGCAGCAAGATGTACACCAAGAG	ATCGCCGGTGCCTTGAC
Redd1	NM_029083.2	CGGGCCGGAGGAAGACT	CTGCATCAGGTTGGCACACA
Murf1	NM_00103904	TGTCTGGAGGTCGTTTCCG	CTCGTCTTCGTGTTCCTTGC
Atrogin-1	NM_026346.2	ACCGGCTACTGTGGAAGAGA	CCTTCCAGGAGAGAATGTGG
Map1lc3B	NM_026160.4	GAGCAGCACCCCACCAAGAT	CGTGGTCAGGCACCAGGAA
Bnip3	NM_009760.4	CCATGTCGCAGAGCGGG	GACGGAGGCTGGAACGC

### Western blot

Western blot was performed according to procedures previously described [15]. In short, frozen gastrocnemius muscle was ground to powder and lysed. Total protein concentration was determined and 12.5 μg was used for Western blotting. The membrane was incubated overnight at 4°C with primary antibodies; S6: #2271, p-S6(Ser235/236): #4856, 4E-BP1: #9452, p-4E-BP1(Ser65): #9451, p-4E-BP1(Thr37/46): #9459, mTOR: #2983, p-mTOR(Ser2448): #2971, p-mTOR(Ser2481): #2974, Akt: #9272, p-Akt(Ser374): #9271, ULK1: #8054, p-ULK1(Ser757): #6888, P62: #5114, TSC2: #4308, p-TSC2(Ser939): #3615, p-TSC2(Thr1462): #3617, TSC2(Ser1387): #5584, AMPK: #2532, AMPK(Thr172): #2531, eIF2α(S51): #3398 (Cell Signaling Technology), eIF2α: AH00802 (Thermo fisher scientific), poly-UB: BMI-PW8805 (ENZO). Blots were probed with a horseradish peroxidase-conjugated secondary antibody and visualized using chemiluminescence in a LAS-3000 Luminescent Image analyzer (Fujifilm). Bands were quantified using the AIDA software (Fujifilm). Ponceau S staining was used to correct for protein loading and transfer [40]. All Western blot data were normalized to abundance of the protein of interest under normoxic conditions without fasting (day 0). Ratios of phosphorylated to total protein are shown and in case total protein levels were not stable between conditions and contributed to changes in phosphorylation ratios it is mentioned in the results section. Poly-Ub conjugation was measured by quantifying total intensity of each lane for signals >200kDa, and corrected for Ponceau-S signal. Western blot samples were randomly loaded; consequently, the images of Western blots were reordered to match the figures.

### Corticosterone measurement

Plasma corticosterone concentrations were determined by HPLC. Cortisol carboxylic acid [41] was added as internal control to plasma samples and corticosterone standards, which were acidified with phosphoric acid. Steroid hormones were subsequently extracted from plasma with diethyl ether and dried with nitrogen gas. The pellet was dissolved in the eluent (acetonitrile/methanol/trifluoroacetic acid) and incubated with sulphuric acid for 20 minutes to convert the steroids into fluorescent products. Subsequently, HPLC analysis was performed as described previously [42]. All corticosterone values were corrected to the internal standard.

### Statistics

Data are shown as means ± SEM. Comparisons were computed with SPSS version 20 (SPSS Inc., IL, USA). Statistical significance between groups was tested by using the Student’s t-test. Differences in fasting response between conditions were statistically tested using a two-way ANOVA. A P-value of <0.05 was considered to be statistically significant and a P value is explicitly stated if 0.05≤P≤0.1.

## Results

### Hypoxia sensitizes skeletal muscle to fasting-induced atrophy

**Fig 1:**
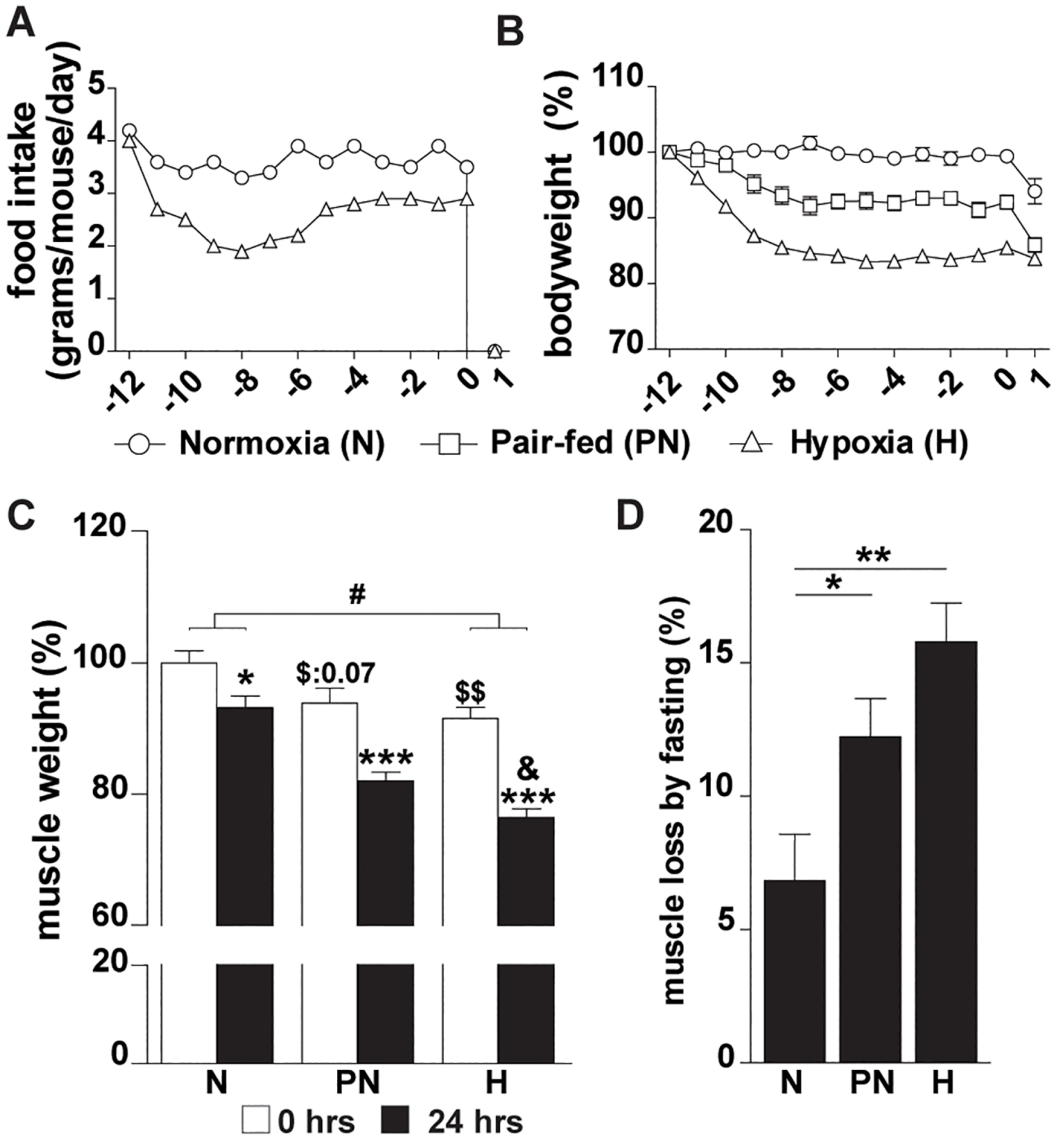
Hypoxia sensitizes skeletal muscle to fasting-induced atrophy. (A): Food intake in gram/mouse/day per experimental condition. (B): Bodyweights. (C): Muscle weights of gastrocnemius muscle normalized to tibia length, were expressed relative to normoxic mice (day 0). (D): Loss of muscle mass per condition after 24 hrs of fasting (expressed relative to mass before fasting; day 0). Significant differences are indicated by (*): comparing 0 hrs fasted and 24 hrs fasted for each condition, * = P< 0.05, ** = P≤ 0.01 or *** = P≤ 0.001. Trends (0.05 ≤ P ≤ 0.1) are indicated by the specific P-value. ($): comparing PN- and H-group to N-group at 0 hrs fasted, (&): comparing PN- and H-group after 24 hrs fasting, (#): comparing the differences in response to fasting between PN- and H-group. (n = 6–8).

Under hypoxic conditions food-intake decreased to ~55% of the normal intake during the first days, and stabilized thereafter at ~81% of normal intake (Fig 1A). The decrease of food intake alone resulted in a stable reduction of bodyweight by 8% as observed in pair-fed normoxic control (PN) compared to normoxic (N) animals (Fig 1B). Hypoxia (H) resulted in ~16% reduction of bodyweight compared to the N-group, which was not due to general growth retardation effects as indicated by equal tibia lengths of the groups (S1 Fig). Subsequent fasting for 24 hrs resulted in an additional ~6% reduction of bodyweight in the N- and PN-group, whereas this was only ~2% in the H-group.

A significant decrease of gastrocnemius muscle weight was observed after 12 days in the H-group (Fig 1C). Fasting for 24 hrs resulted in muscle weight loss in control, pair-fed and hypoxic animals (Fig 1C and 1D) and was most pronounced in the H-group (Fig 1D). The response to fasting in the H-group was different compared to the N-group and final muscle weight was significantly lower than muscle weight of fasted animals in the PN-group. Overall, hypoxia sensitized skeletal muscle to fasting-induced atrophy, which is mainly accounted for by a hypoxia-induced reduction in food-intake. To distinguish the specific effects of hypoxemia from those due to reduced food intake evoked by hypoxia, the response to fasting was compared between the H- and PN-groups, and expressed relative to normoxic baseline levels in the subsequent analyses.

### Hypoxia blunts fasting-induced protein degradation signaling

**Fig 2:**
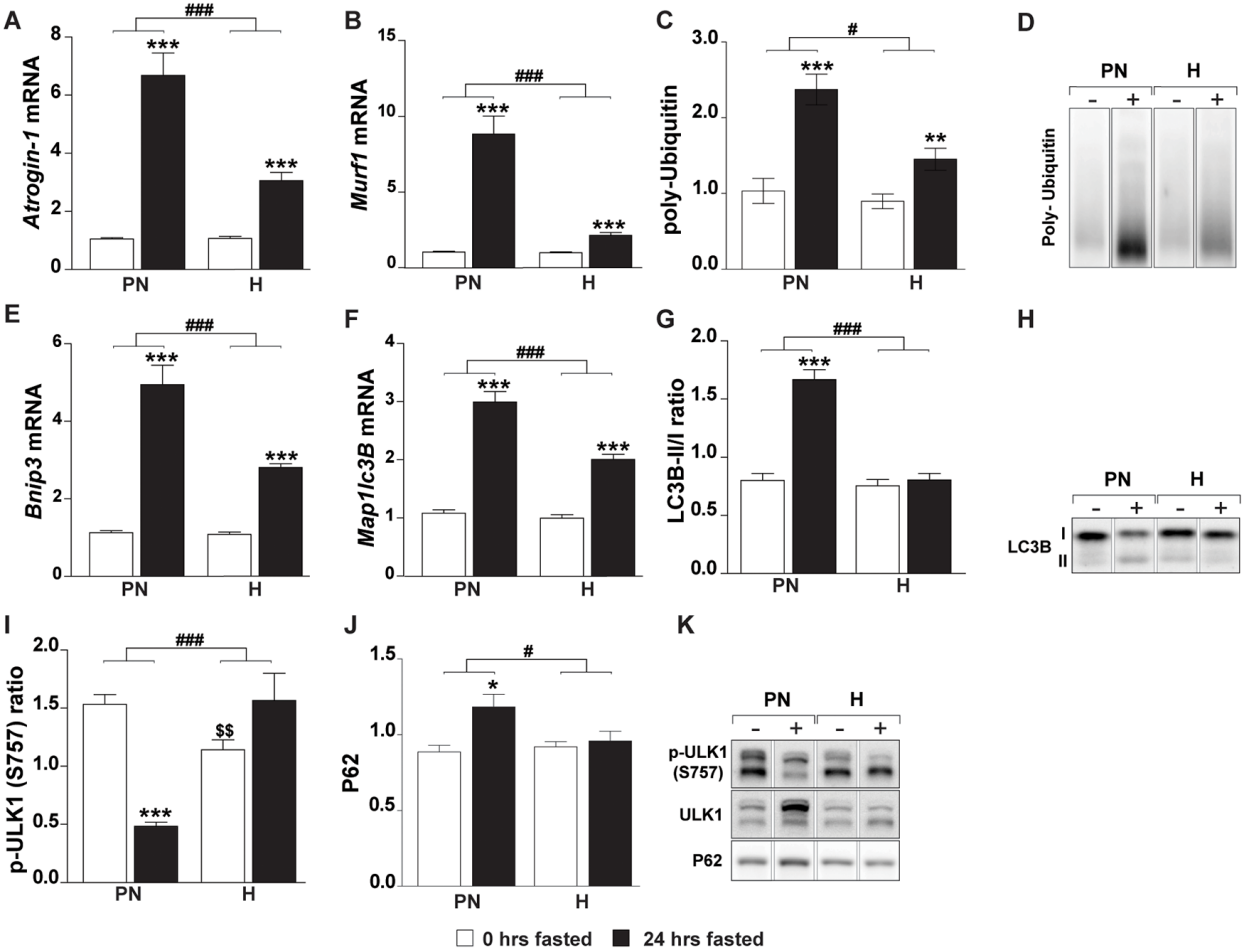
Hypoxia blunts fasting-induced protein degradation signaling. Expression of UPS-associated genes: (A) *Atrogin-1*, (B) *Murf1*. (C) Total poly-ubiquitin. (D) Representative image of poly ubiquitin blot Western blot (reordered to match figures). Expression of ALP-associated genes: (E) *Bnip3*, (F) *Map1lc3B*. (G) Protein ratio of LC3B-II over I. (H) Representative image of LC3B Western blot (reordered to match figures). (I) Protein ratio of p-ULK1 S757 over total ULK1, (J) total P62 protein, (K) representative images of ULK1 and P62 Western blots (reordered to match figures). Data are normalized to the basal expression of the normoxic group at day 0. Significant differences are indicated by (*): comparing 0 hrs fasted and 24 hrs fasted for each condition, * = P< 0.05, ** = P≤ 0.01 or *** = P≤ 0.001. Trends (0.05 ≤ P ≤ 0.1) are indicated by the specific P-value. ($): comparing PN- and H-group to N-group at 0 hrs fasted, (#): comparing the differences in response to fasting between PN- and H-group. (n = 6–8).

In order to investigate activation of the protein degradation machinery, expression of effectors of the ubiquitin 26S-proteasome system (*Atrogin-1*, *Murf1*), levels of conjugated poly-ubiquitin and the autophagy lysosomal pathway (*Bnip3*, *Map1lc3B*) were measured. Fasting increased gene expression of *Atrogin-1*, *Murf1*, *Bnip3* and *Map1lc3B* in the PN-group (Fig 2A, 2B, 2E and 2F). However, despite more extensive muscle atrophy, hypoxia significantly blunted the fasting-induced expression of these same genes. Fasting induced poly-ubiquitin conjugation was attenuated in hypoxic muscle (Fig 2C and 2D), confirming blunted activation of the ubiquitin proteasome system suggested by the E3 ligase expression. To substantiate activation of the autophagic machinery LC3B-I to LC3B-II levels were investigated (Fig 2G and 2H), LC3B II/I ratio was elevated in the PN-group resulting from reduced LC3B-I (0.6 fold) and increased LC3B-II (1.3 fold) amounts. In contrast, no changes in LC3B-I, -II or ratios were found in the H-group. The UNC51-like kinase 1 (ULK1) induces autophagy through interaction with AMPK. This interaction is inhibited by mTORC1 through phosphorylation at Ser757 [43]. ULK1 S757 phosphorylation ratio (Fig 2I and 2K) was decreased by fasting in the PN-group as a result from increased ULK1 protein amounts (2.1 fold) combined with a decrease of ULK1 S757 phosphorylation (0.7 fold). Downstream of autophagy initiation (ULK) and autophagosome formation (LC3B ratio), P62 (Fig 2J and 2K) abundance was measured. P62 was increased by fasting in the PN-group, but not in the H-group. These data suggests that hypoxia prevents fasting-induced activation of autophagy.

### Hypoxia attenuates the ISR and blocks fasting-induced inhibition of mTORC1

**Fig 3:**
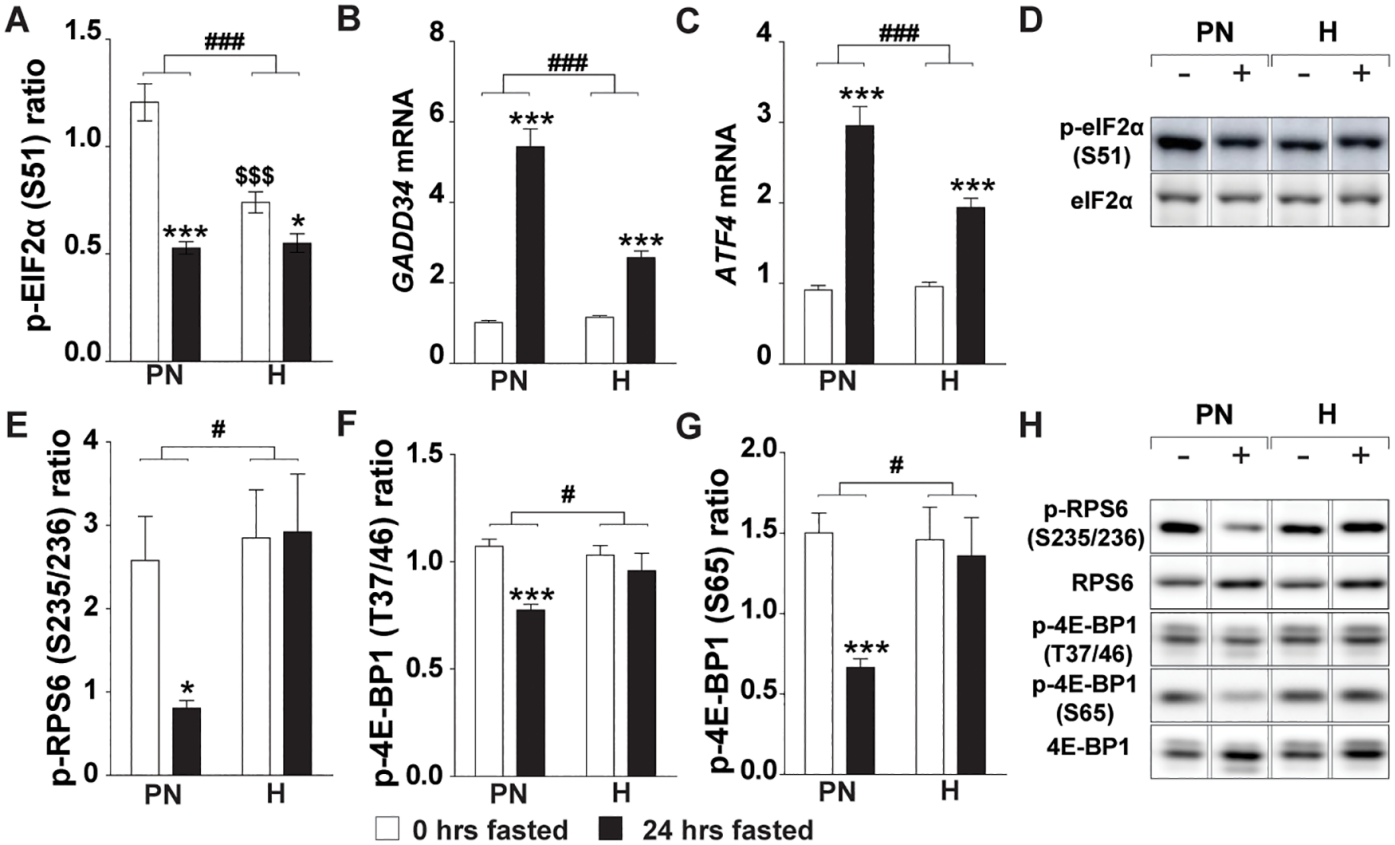
Hypoxia attenuates the ISR and blocks fasting-induced inhibition of mTORC1. (A) Ratio of p-eIF2α (S51) over total eIF2 α. Expression of ISR-associated genes: (B) *Gadd34*, (C) *Atf4*. (D) Representative images of eIF2α Western blots (reordered to match figures). (E) Ratio of p-RPS6 (S235/236) over total RPS6. (F) Ratio of p-4E-BP1 (T37/46) over total 4E-BP1. (G) Ratio of p-4E-BP1 (S65) over total 4E-BP1. (H) Representative images of Western blots (reordered to match figures). Data are normalized to the basal expression of the normoxic group at day 0. Significant differences are indicated by (*): comparing 0 hrs fasted and 24 hrs fasted for each condition, * = P< 0.05, ** = P≤ 0.01 or *** = P≤ 0.001. Trends (0.05 ≤ P ≤ 0.1) are indicated by the specific P-value. ($): comparing PN- and H-group to N-group at 0 hrs fasted, (#): comparing the differences in response to fasting between PN- and H-group. (n = 6–8).

The integrated stress response renders the protein synthesis machinery non-permissive to upstream signaling cues, and its activating kinases culminate in the phosphorylation of eIF2alpha [39]. Baseline eIF2alpha phosphorylation was reduced by hypoxia, and although 24h fasting resulted in suppression of eIF2alpha phosphorylation in normoxic and hypoxic muscle, the magnitude of the response was significantly different (Fig 3A and 3D). GADD34 is a downstream target of the ISR with phosphatase activity directed towards eIF2a [39]. GADD34 baseline expression was not affected by hypoxia, and its mRNA transcript abundance was increased following fasting, although this response was significantly attenuated in hypoxic muscle (Fig 3B). A similar ATF4 expression pattern was observed (Fig 3C), suggesting fasting-induced activation of downstream ISR effectors is attenuated by hypoxia.

Pre-conditioning by hypoxia or pair-feeding resulted in an increased basal mTORC1 activity which was similar prior to fasting in hypoxia and pair-feeding groups compared to control, as illustrated by their equally increased ratios of phosphorylated over total ribosomal protein S6 (RPS6) and eukaryotic translation initiation factor 4E binding protein (EIF4EBP1, a.k.a. 4E-BP1), respectively (Fig 3E, 3F, 3G and 3H). As expected, fasting resulted in a reduction of the ratio of RPS6 and 4E-BP1 phosphorylation in the PN group. Surprisingly, relative phosphorylation levels were retained following fasting under hypoxia. Total protein levels did not account for this altered response to fasting as total 4E-BP1 protein concentration increased equally in the PN-group (1.2 fold) and H-group (1.3 fold) and no differences were found in RPS6. This altered response in RPS6 and 4E-BP1- as well as ULK1 S757 (Fig 2I) phosphorylation, imply that fasting-induced inhibitory signaling to reduce mTORC1 activity is disabled under hypoxic conditions.

### mTORC1 activity is uncoupled from AKT1/TSC2 and AKT1/mTOR signaling under hypoxic conditions

**Fig 4:**
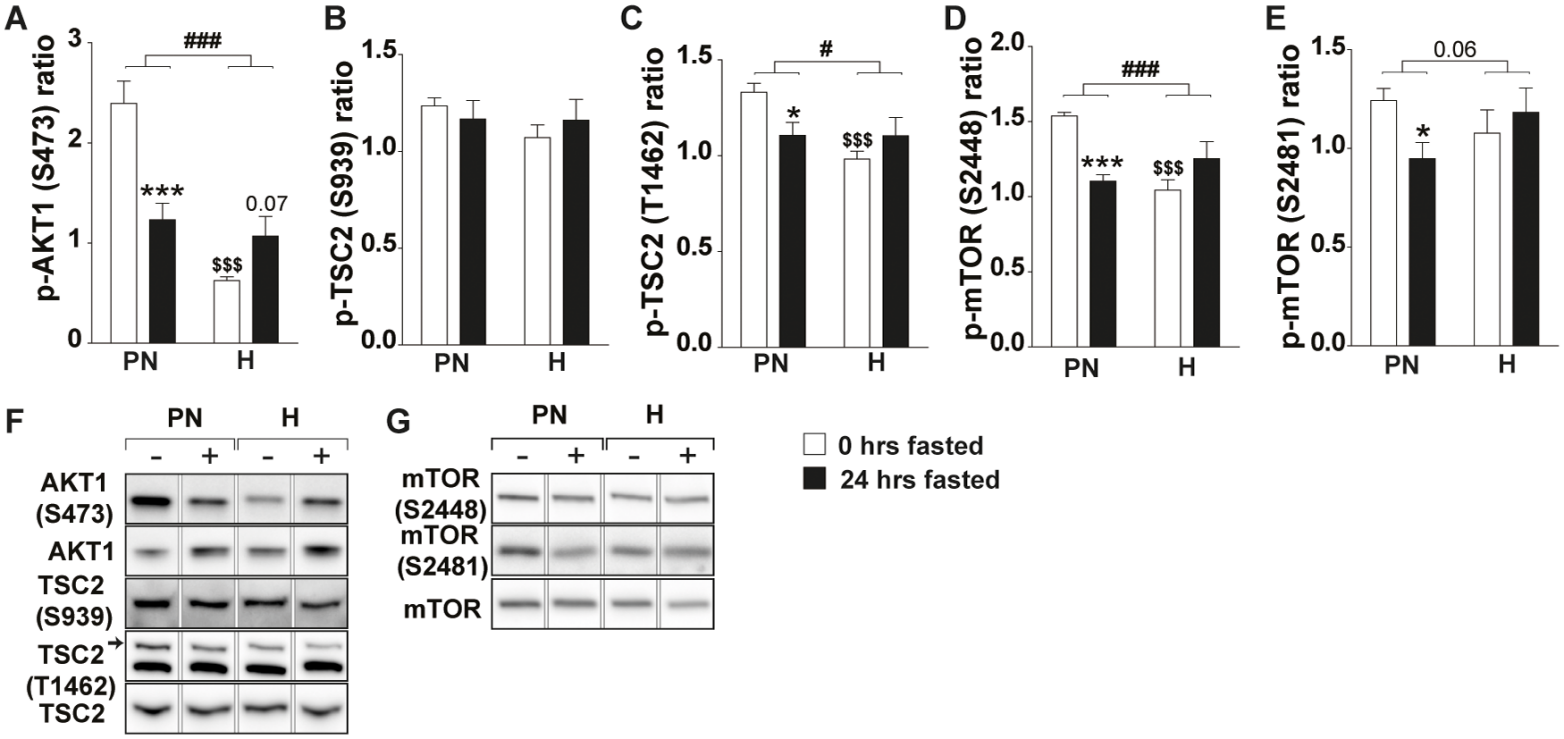
Fasting-induced attenuation of AKT1/TSC2 and AKT1/mTORC1 signaling does not correlate with mTORC1 activity under hypoxic conditions. (A) Ratio of p-AKT1 (S473) over total AKT1. (B) Ratio of p-TSC2 (S939) over total TSC2. (C) Ratio of p-TSC2 (T1462) over total TSC2. (D) Ratio of p-mTOR (S2448) over total mTOR. (E) Ratio of p-mTOR (S2481) over total mTOR. Representative images of Western blots (reordered to match figures). Data are normalized to the basal expression of the normoxic group at day 0. Significant differences are indicated by (*): comparing 0 hrs fasted and 24 hrs fasted for each condition, * = P< 0.05, ** = P≤ 0.01 or *** = P≤ 0.001. Trends (0.05 ≤ P ≤ 0.1) are indicated by the specific P-value. ($): comparing PN- and H-group to N-group at 0 hrs fasted, (#): comparing the differences in response to fasting between PN- and H-group. (n = 6–8).

mTORC1 activity is controlled by multiple upstream signaling pathways, including inhibition of the TSC1/2 complex [44] through AKT mediated phosphorylation of TSC2 on sites S939 and T1462 [45, 46]. Pre-conditioning increased AKT1 S473 phosphorylation in the PN-group, which was significantly lower in the H-group (Fig 4A and 4F). Subsequent fasting resulted in reduced AKT1 phosphorylation in the PN-group, whereas under hypoxic conditions an opposite trend (p = 0.07) towards increased phosphorylation was observed. TSC2 S939 phosphorylation did not differ between the PN- and H-groups and did not change by fasting (Fig 4B and 4F). In contrast, TSC2 T1462 phosphorylation was clearly reduced by hypoxia, and by fasting in the PN-group (Fig 4C and 4F). However, fasting did not further decrease TSC2 T1462 phosphorylation in hypoxic mice. AKT1 also phosphorylates mTOR at S2448, which may be followed by mTOR S2481 autophosphorylation [47]. In line with AKT1 S473 phosphorylation levels, the basal phosphorylation of mTOR S2448 and S2481 were increased in the PN-group, whereas basal levels of mTOR S2448 phosphorylation in the H-group were lower (Fig 4D, 4E and 4G). Fasting reduced mTOR S2448 and S2481 phosphorylation in the PN- but not the H-group. Overall, hypoxia suppressed basal AKT1/TSC2 and AKT1/mTOR signaling, and fasting had no additional effect. This implied that other upstream regulatory cues are involved in the lack of mTORC1 inactivation upon starvation under hypoxic conditions.

### Fasting-induced AMPK activity is suppressed by hypoxia

**Fig 5:**
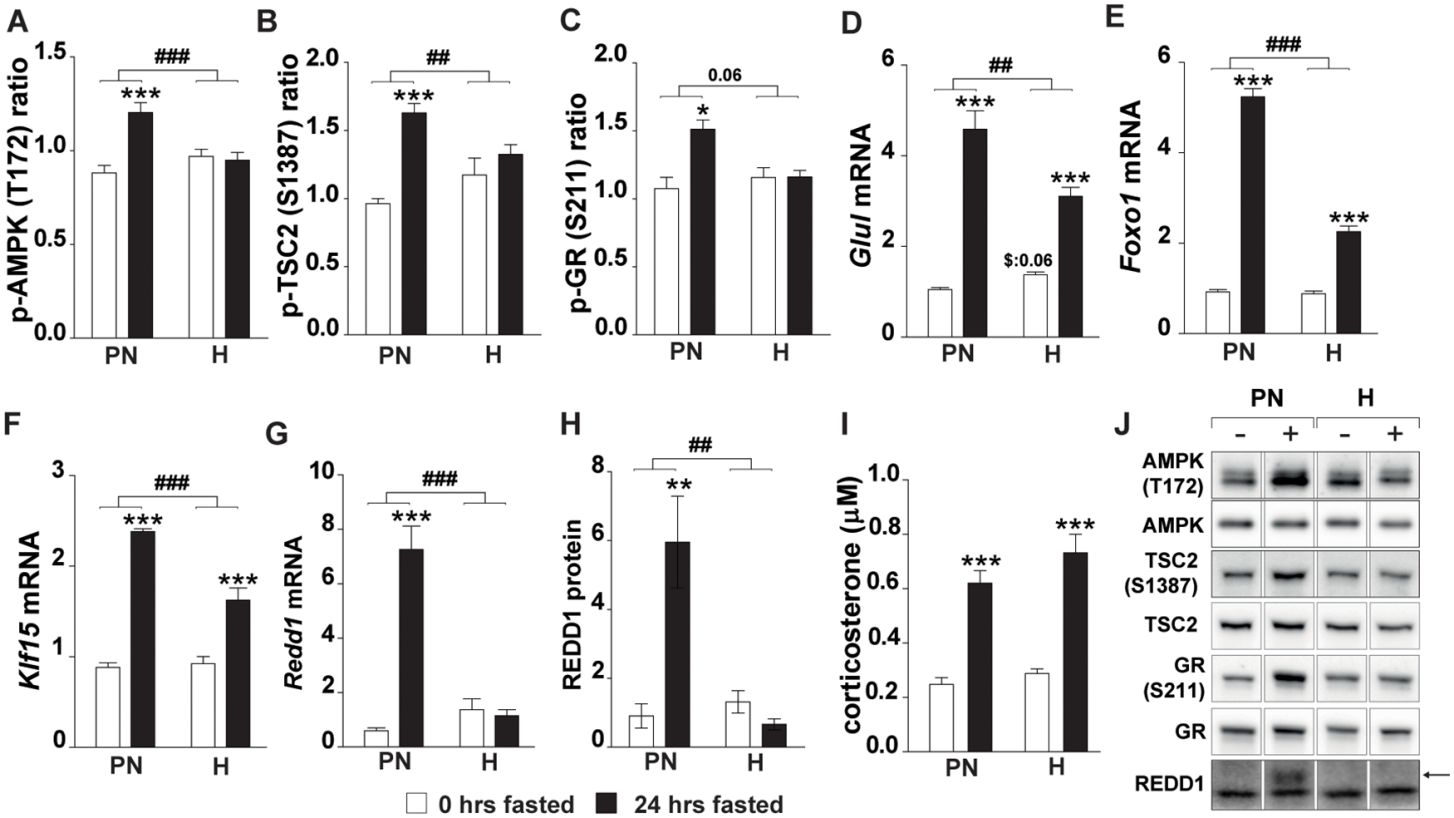
Fasting-induced control of mTORC1 activity by AMPK/TSC2 signaling is altered by hypoxia. (A) Ratio of p-AMPK (T172) over total AMPK. (B) Ratio of p-TSC2 (S1387) over total TSC2 (for total TSC2 see Fig 5B panel 6). (C) Ratio of p-GR (S211) over total GR. mRNA expression of GR target genes: (D) *Glul*, (E) *Foxo1*, (F) *Klf15*, (G) *Redd1*. (H) Redd1 protein. (I) Plasma corticosterone concentration (J) Representative images of Western blots (reordered to match figures). Data are normalized to the basal expression of the normoxic group at day 0. Significant differences are indicated by (*): comparing 0 hrs fasted and 24 hrs fasted for each condition, * = P< 0.05, ** = P≤ 0.01 or *** = P≤ 0.001. Trends (0.05 ≤ P ≤ 0.1) are indicated by the specific P-value. ($): comparing PN- and H-group to N-group at 0 hrs fasted, (#): comparing the differences in response to fasting between PN- and H-group. (n = 6–8).

mTORC1 activity can be inhibited through AMPK signaling following TSC2–activatiing S1387 phosphorylation by AMPK [35]. Fasting increased AMPK T172 phosphorylation in the PN-group but not in hypoxic mice (Fig 5A and 5J). Basal TSC2 S1387 phosphorylation levels were similar to control (N) in both the PN- and the H-group (Fig 5B and 5J). Fasting resulted in an increased phosphorylation of TSC2 S1387 in the PN- but not the H-group, corresponding to the changes observed in the AMPK T172 phosphorylation. As key regulator of energy balance, AMPK is involved in protein synthesis signaling as well as protein degradation through activation of p38 MAPK and subsequent phosphorylation of the glucocorticoid receptor (GR) at site S211 [38, 48]. Increased GR phosphorylation was found by fasting under normoxic conditions, but not under hypoxic conditions (Fig 5C and 5J). Accordingly, increased mRNA levels of GR transcriptional target genes (*Glul*, *Foxo1*, *Klf15* and *Redd1*) and REDD1 protein confirmed elevated GR activity in response to fasting under normoxic conditions, which was blunted under hypoxic conditions (Fig 5D, 5E, 5F, 5G, 5H and 5J). To probe whether reduced corticosterone levels under hypoxic conditions contributed to attenuated GR activation, corticosterone (cc) levels were measured in plasma (Fig 5I). Fasting increased corticosterone levels equally in the PN- and H-group. Overall, altered responsiveness of AMPK to fasting cues under hypoxia corresponds to retained protein synthesis signaling and attenuated proteolysis activation via TSC2 control of mTORC1 activity, and GR-regulation of UPS and ALP gene expression, respectively.

## Discussion

**Fig 6:**
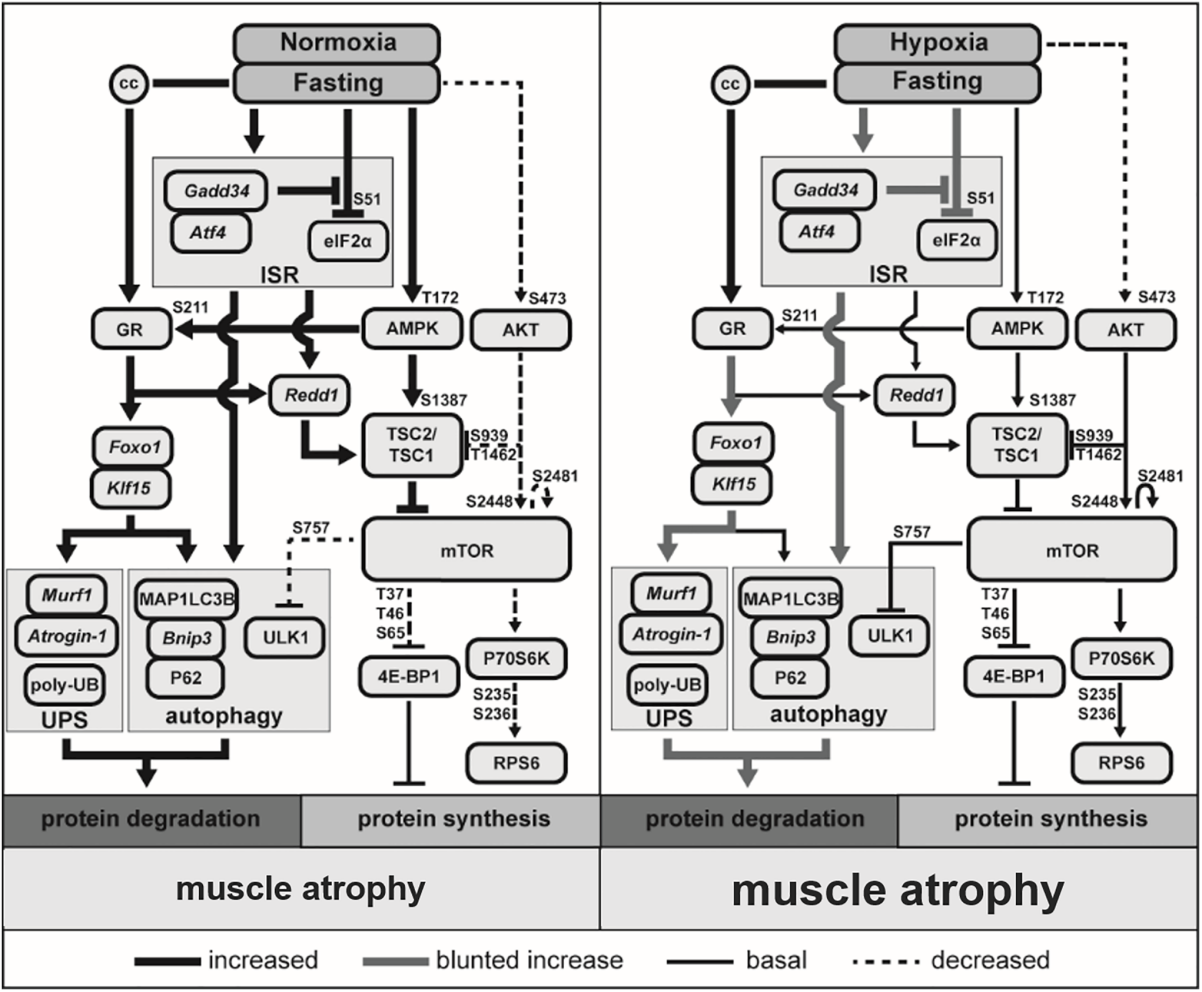
The effects of hypoxia and fasting on protein turnover signaling. Targets measured in this study by mRNA expression are indicated by italics, proteins by capital letters, and corticosterone is indicated by the abbreviation cc.

Our study shows a more pronounced loss of muscle mass after fasting in hypoxic compared to normoxic animals. Hypoxia blunts fasting-induced activation of protein degradation and autophagy signaling, which corresponds with decreased GR S211 phosphorylation and GR-mediated gene expression, suggesting reduced activation of this regulator of muscle proteolysis under hypoxia. In addition, hypoxia blunts fasting-induced activation of the integrated stress response, which may affect inhibitory control of protein synthesis following fasting. In line, fasting-induced inhibition of mTORC1 activity is absent under hypoxic conditions. Correspondingly, the AMPK/TSC2-mediated control of mTORC1 activity appears disabled, as under hypoxic conditions the fasting-induced phosphorylation of AMPK (T172) and AMPK-mediated S1387 phosphorylation of TSC2 are absent. The findings of this study on muscle protein turnover regulation in response to fasting are summarized in Fig 6.

Previously, we and others showed that 4 and 21 days of hypoxia results in loss of muscle mass which was partially due to reduced food intake evoked by hypoxia [13–15]. Similarly, in the current study, 12 days of hypoxia or pair-feeding under normoxia induce muscle loss. Fasting leads to a significantly larger muscle loss in the hypoxic group compared to the normoxic group, and this is mainly explained by the reduction in food intake prior to fasting. The relative preservation of body weight of hypoxic animals in response to fasting may reflect conservation of organ and tissue mass other than skeletal muscle, resulting from an adaptive response to the reduction in body weight that developed during 12 days of preceding hypoxia. In line with previous studies by other groups [22, 23, 49, 50], the expression of ALP and UPS-related genes is increased by fasting under normoxic conditions. Surprisingly, despite the accentuated muscle mass loss, hypoxia attenuates fasting-induced increases of mRNA transcript levels of UPS and ALP effectors. In this study proteolytic activity was not measured, but it has been shown previously, that increased expression of these effectors of proteolysis is accompanied by increased protein degradation in response to fasting [51, 52]. In line, poly-Ub conjugation as the preceding step of UPS-mediated proteolysis is elevated in muscle of fasted animals, while hypoxia strongly attenuates the magnitude of this fasting-induced response. Moreover, based on the ULK1 and LC3B data, autophagy signaling is upregulated by fasting but not under hypoxic conditions. This may result from the attenuated ISR activation by fasting in hypoxic muscle, as ATF4 has been implicated in autophagy induction [39], although activation of autophagy in skeletal muscle following ISR inhibition has also been demonstrated [53]. Alternatively, *Klf15* [30, 54] and *Foxo1* [21, 32, 55] have been implicated in the regulation of the ALP and UPS responses, and are targets of GR signaling. Since the fasting-induced increase of the expression of *Klf15* and *Foxo1* is blunted under hypoxic conditions, altered GR-mediated gene expression by hypoxic stress may be responsible for the observed attenuation of the proteolytic response. A blunted response of KLF15 to dexamethasone is also observed in heat stressed C2C12 myotubes [56], indicating a change in GR mediated signaling following stress such as heat or hypoxic pre-conditioning. GR transcriptional activity is regulated at multiple levels, including AMPK-mediated phosphorylation at GR at serine 211 [38]. As reduced expression of GR-sensitive genes, including those of the proteolytic machinery in response to fasting under hypoxia corresponds to attenuated AMPK activity and absence of GR S211 phosphorylation, this suggests that fasting-induced activation of an AMPK-GR regulated transcriptional program to increase muscle proteolysis is disabled by hypoxia.

While fasting inhibits muscle mTORC1 activity in pair-fed animals under normoxic conditions as expected [52, 57, 58], mTORC1 activity is retained in muscle of fasted animals under hypoxia. This is in line with a previous report by our group [15], in which acute hypoxia (4 days) blocked the inhibition of mTORC1 in response to reduced food intake. REDD1 can inhibit mTORC1 activity by preventing binding of TSC2 to 14-3-3 [34]. The upregulation of *Redd1* expression and protein levels in response to fasting under normoxia is in line with observations in fasted muscle by others [59]. Interestingly, the absence of fasting-induced expression of *Redd1* and protein levels during hypoxia correlates with retained mTORC1 activity as assessed by the 4E-BP1 and S6 phosphorylation status. This suggests that altered REDD1 regulation by hypoxia contributes to the disabled control of mTORC1 activity. Transcriptional regulation of REDD1 by ATF4 as a downstream effector of the ISR has been proposed to extend the acute inhibitory effects of ISR-induced eIF2alpha phosphorylation on protein synthesis [60]. Accordingly, the induction of ATF4 transcripts following fasting, and its attenuation in hypoxic muscle correlates with REDD1 expression, implying inadequate activation of the ISR in impaired suppression of mTORC1 activity in fasted, hypoxic muscle. Interestingly, altered REDD1 regulation and control of mTORC1 activity was also reported for glucocorticoid-induced suppression of anabolic signaling following heat stress preconditioning of myotubes [56]. Moreover, *Redd1* is considered a GR-responsive gene, required for glucocorticoid induced muscle atrophy [61]. Since *Redd1* expression and protein levels mirror the GR-sensitive gene expression patterns, this suggests that the hypoxia-imposed lack of AMPK activation and subsequent GR S211 phosphorylation may not only affect transcriptional regulation of proteolysis but also protein synthesis.

Insulin and insulin-like growth factor-1 are known to stimulate mTORC1 activity via the AKT1/TSC2 and AKT1/mTORC1 pathways. Phosphorylated active AKT1 deactivates TSC2 through phosphorylation at S939 and T1462, which results in binding of TSC2 to 14-3-3 [45, 46]. The fasting-induced reduction of AKT1 phosphorylation observed in PN has been previously reported under normoxic conditions [31], and corresponds to the fasting-induced decrease of serum insulin [62, 63]. 4 weeks of hypoxia also reduces serum insulin concentrations in mice [64], which may explain the reduced AKT1 phosphorylation observed in the fed state after 12 days of hypoxia in this study. However, as mTORC1 activity is preserved in the fed and fasted state under hypoxic conditions, this does not correspond to lowered AKT phosphorylation. Therefore alterations in AKT1/TSC2 and AKT1/mTORC1 signaling cannot be responsible for the deregulation of mTORC1 activity under hypoxic conditions.

AMPK is a sensor of the energy status of a cell [65]. Upon ATP depletion AMPK can suppress mTORC1 activity through activation of TSC2 by phosphorylation of S1387 and T1227 [35]. Although hypoxia has been shown to increase AMPK activity *in vitro* [66, 67], in our mouse model basal AMPK phosphorylation was not increased after 12 days of hypoxia or by fasting under hypoxic conditions. These conflicting findings on the effects of hypoxia on AMPK activity probably reflect an interaction of hypoxia with systemic effects absent *in vitro*, such as elevated corticosterone levels. Under normoxic conditions, fasting results in increased AMPK phosphorylation and inhibition of mTORC1 activity. However, under hypoxic conditions, fasting does not increase AMPK phosphorylation. Lack of AMPK activation by hypoxia has also been demonstrated in older mice (24 months), although there basal levels were already higher [68]. Hypoxia results in elevated levels of reactive oxygen species (ROS) in skeletal muscle [69] and it has been demonstrated that ROS production can block fasting-mediated AMPK activation [70]. In addition, AMP accumulation is required for most forms of AMPK activation. Both elevated IMP but unchanged AMP, ADP and ATP levels [71], as well as increased AMP degradation to IMP and ammonia during exercise have been shown in patients with advanced COPD [72]. Moreover, hypoxemia (11% ambient O_2_) resulted in increased IMP concentrations without altering AMP levels in human skeletal muscle during submaximal exercise [73]. Combined with our findings, this suggests that a lack of AMP accumulation due to increased conversion to IMP under hypoxia may impair AMPK activation and subsequent inhibition of mTORC1 in response to fasting. Interestingly, muscle intrinsic aberrations such as observed in Myotonic dystrophy type I may also interfere with AMPK activation and control of protein synthesis in skeletal muscle. In a mouse model of Myotonic dystrophy type I, fasting-induced activation of AMPK was absent and accompanied with sustained protein synthesis signaling, similar to our findings [74]. Interestingly, pharmacological activation of AMPK not only corrected protein synthesis signaling, but also reduced myotonia in this model, illustrating the importance of appropriate AMPK signaling in muscle homeostasis. Similarly, inhibition of the ISR, which could be expected to facilitate anabolic signaling, exacerbates muscle wasting in cancer cachexia [74]. Collectively, this data suggests that ISR and AMPK activation in response fasting is strongly attenuated by hypoxia, resulting in altered proteolytic signaling and disabled suppression of synthesis signaling (Fig 6). Although the downstream cellular effectors of additional muscle loss require further elucidation, the critical role of appropriate AMPK and ISR regulation in muscle homeostasis and atrophy observed in other studies [53, 74], suggests their impaired activation during hypoxia may aggravate fasting induced muscle atrophy. Subsequent studies should investigate ISR, AMPK and GR activation in relevant models and skeletal muscle of subjects with hypoxemia, including COPD patients, preferentially in combination with protein synthesis and proteolysis measurements, to further elucidate the regulation of protein turnover and muscle wasting in response to hypoxia.

## Conclusion

Hypoxia aggravates fasting-induced muscle atrophy and suppresses AMPK and ISR activation. Altered AMPK-mediated regulation of mTORC1 and GR may underlie aberrant protein turnover signaling and affect muscle atrophy responses in hypoxic skeletal muscle.

## Supporting information

S1 Fig Tibia length. Tibia length after 12 days of preconditioning.
(TIF)
