# Supplementary figures and images for: Hypoxia impairs adaptation of skeletal muscle protein turnover- and AMPK signaling during fasting-induced muscle atrophy
Source: PLoS One. 2018 Sep 13;13(9):e0203630. doi: 10.1371/journal.pone.0203630 (PMC6136752; doi:10.1371/journal.pone.0203630)

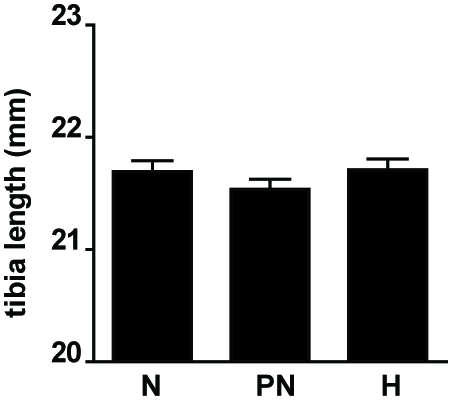

Supplement: S1 Fig — Tibia length after 12 days of preconditioning. (TIF) [file pone.0203630.s001.tif]
